# Dermoscopic Profile of Melasma: A Cross-Sectional Observational Study From a Tertiary Care Center in North India

**DOI:** 10.7759/cureus.112574

**Published:** 2026-07-13

**Authors:** Amrit Pant, Ram Chander, Sania Khan, Vijay K Garg, Somya Sharma, Gulshant Panesar, Shivangi Singh

**Affiliations:** 1 Dermatology, Santosh University, Ghaziabad, IND; 2 Dermatology, Vinayak Skin Clinic, New Delhi, IND

**Keywords:** dermatoscopy, dermoscopy, melanoses, melasma, quality of life

## Abstract

Background

Melasma is an acquired, chronic pigmentary disorder affecting sun-exposed areas of the face, predominantly in women of reproductive age. Dermoscopy has emerged as a non-invasive, in vivo imaging tool that can delineate the characteristic features of melasma, assess pigment depth, and facilitate differentiation from other facial melanoses. However, systematic dermoscopic data from Indian populations remain limited. This study aimed to describe the dermoscopic profile of melasma and study dermoscopic findings with clinical parameters, Wood’s lamp-based pigment depth, disease severity (modified Melasma Area and Severity Index (mMASI)), and quality of life (Hindi version of the Melasma Quality of Life Scale (Hi-MelasQoL)).

Methodology

This hospital-based, observational, cross-sectional study enrolled 119 patients with clinically diagnosed melasma attending the dermatology outpatient department of a tertiary care institution. All patients underwent detailed clinical profiling, Wood’s lamp examination for depth assessment, dermoscopy using the DermLite DL5 dermatoscope, mMASI scoring, and quality of life evaluation using the validated Hi-MelasQoL.

Results

The study cohort had a mean age of 34.31 ± 7.65 years with a female-to-male ratio of 4.1:1. The most prevalent dermoscopic features were globules (N = 116, 97.5%), pseudoreticular enhancement (N = 114, 95.8%), perifollicular sparing (N = 66, 55.5%), telangiectasias (N = 61, 51.3%), arcuate pattern (N = 50, 42.0%), and dots/granules (N = 27, 22.7%). The mean mMASI was 4.25 ± 1.99, and the mean Hi-MelasQoL was 29.93 ± 10.22. A strong positive correlation was found between mMASI and Hi-MelasQoL (Spearman’s rho = 0.74, p < 0.001).

Conclusions

Pseudoreticular enhancement and globules are the dominant dermoscopic hallmarks of melasma in the Indian population, confirming findings from prior studies. The frequent co-occurrence of telangiectasias underscores the vascular component of melasma pathogenesis and has direct therapeutic implications. Dermoscopy is a valuable tool for diagnostic confirmation, depth assessment, and treatment guidance in melasma.

## Introduction

Melasma is an acquired, chronic pigmentary disorder predominantly in women of reproductive age, characterized by symmetrically distributed hyperpigmented macules and patches on sun-exposed facial areas, including the forehead, cheeks, nose, upper lip, and chin [1,2]. Its pathogenesis involves a complex mix of genetic predisposition, chronic ultraviolet (UV) radiation exposure, female sex hormones, and local cutaneous factors such as increased vascularity and dermal elastosis [1,3].

The clinical diagnosis of melasma is primarily based on visual inspection. However, objective assessment of pigment depth and disease severity has traditionally relied on Wood’s lamp examination, a method with recognized limitations in individuals with darker skin phototypes [4].

Over the past decade, dermoscopy has gained increasing acceptance as a non-invasive, in vivo imaging adjunct for evaluating pigmentary disorders of the skin [5,6]. By providing real-time magnification and illumination of both the epidermis and superficial dermis, dermoscopy allows characterization of the distribution and morphology of pigmentation, assessment of vascular structures, and differentiation of melasma from mimicking conditions such as exogenous ochronosis, drug-induced hyperpigmentation, and Hori’s nevus [5-7].

The reported dermoscopic features of melasma include an accentuated pseudoreticular pigmented network, reticuloglobular pattern, dotted or granular pigmentation, arcuate structures, perifollicular sparing, and vascular patterns including telangiectasias [7-9]. Several studies have described these features in diverse ethnic cohorts; however, systematic data from the Indian subcontinent, where melasma constitutes a major burden of pigmentary disease in routine dermatological practice, remain relatively limited [7,9].

The modified Melasma Area and Severity Index (mMASI) is the most widely used validated instrument for objective severity assessment, while the Hindi version of the Melasma Quality of Life Scale (Hi-MelasQoL) enables psychosocial impact evaluation in the Indian context [10,11]. Understanding the interplay between dermoscopic features, clinical phenotype, and quality of life impairment is essential for devising comprehensive management strategies.

This study aimed to characterize the dermoscopic profile of melasma in a cohort of patients from a tertiary care institution in North India and to evaluate dermoscopic findings alongside clinical parameters, including distribution pattern, pigment depth assessed by Wood’s lamp, disease severity, and quality of life impairment.

## Materials and methods

Study design and setting

A hospital-based, cross-sectional, observational study was undertaken in the Department of Dermatology, Venereology and Leprosy at Santosh Medical College and Hospital, Ghaziabad, Uttar Pradesh, India. The research spanned one and a half years and obtained ethical approval from the Institutional Ethics Committee of Santosh Deemed to Be University.

Sample size and participants

The sample size was calculated using the standard formula for prevalence studies (N = Z²p(1-p)/E²), with a reported prevalence of 8.4% for melasma, a confidence level of 95%, and a margin of error of 5%, yielding a minimum required sample size of 119 patients. Consecutive patients above 18 years of age with a clinical diagnosis of melasma attending the outpatient clinic were enrolled after obtaining written informed consent.

Inclusion and exclusion criteria

Patients of either sex above 18 years of age with clinically diagnosed melasma were included. Patients below 18 years, pregnant or lactating women, those with active cutaneous infection, those with known hypersensitivity to topical anesthetics, those on anticoagulant therapy, and those who had received any treatment for melasma within the preceding month were excluded.

Clinical assessment

All enrolled patients underwent a clinical evaluation, including assessment of age of onset, disease duration, Fitzpatrick skin type, distribution pattern (malar, centrofacial, or mandibular), associated hormonal and reproductive factors in women (oral contraceptive pill use, menstrual irregularity, pregnancy-related onset), family history, and thyroid disease. The mMASI, based on assessment of the area of involvement (A) and darkness of hyperpigmentation (D) across four facial regions (forehead, right malar, left malar, and chin), was calculated at baseline [10]. Depth of pigmentation was classified as epidermal, mixed, or dermal using Wood’s lamp examination. Quality of life impairment was assessed using the validated Hi-MelasQoL, a 10-item questionnaire evaluating the impact of melasma on emotional well-being, social interactions, and daily activities [11].

Dermoscopic evaluation

Polarized dermoscopy was performed using a DermLite DL5 dermatoscope (10× magnification), and images were captured with an iPhone 15 Pro. For each patient, six images were obtained from the affected area. Dermoscopic findings were interpreted and recorded according to patterns previously described in the literature. Dermoscopic images were systematically evaluated for the following pre-specified features: pseudoreticular enhancement (accentuation of the normal pseudoreticular pigmentary network corresponding to follicular openings and epidermal pigmentation), globules (discrete round-to-oval hyperpigmented structures), dots (small brownish pigmentary deposits), arcuate structures (curved pigmented streaks), perifollicular sparing (hypopigmented halos around follicular openings), and telangiectasias (fine linear or arborizing blood vessels).

Statistical analysis

Data were entered in Microsoft Excel (Microsoft Corp., Redmond, WA, USA) and analyzed using statistical software. Continuous variables are expressed as mean ± standard deviation (SD) or median (interquartile range (IQR)) as appropriate. Categorical variables are presented as frequencies and percentages with 95% confidence intervals (CIs). The association between Hi-MelasQoL and mMASI was evaluated using Spearman’s rank correlation coefficient, given the non-normal distribution of at least one variable. Univariable linear regression was performed with Hi-MelasQoL as the dependent variable. A p-value <0.05 was considered statistically significant throughout.

## Results

Demographic and clinical profile

A total of 119 patients with clinically diagnosed melasma were enrolled. The demographic and clinical characteristics of the study cohort are summarized in Table 1.

**Table 1:** Demographic and clinical characteristics of the study cohort (n = 119). mMASI = modified Melasma Area and Severity Index; Hi-MelasQoL = Hindi version of the Melasma Quality of Life Scale; SD = standard deviation; CI = confidence interval

Characteristic	n (%)	95% CI
Age, mean ± SD (years)	34.31 ± 7.65	—
Sex (female)	96 (80.7%)	72.2–87.1
Malar pattern	65 (54.6%)	45.3–63.7
Centrofacial pattern	54 (45.4%)	36.3–54.7
Epidermal depth (Wood’s lamp)	64 (53.8%)	44.4–62.9
Mixed depth	52 (43.7%)	34.7–53.1
Dermal depth	3 (2.5%)	0.7–7.7
mMASI, mean ± SD	4.25 ± 1.99	—
Hi-MelasQoL, mean ± SD	29.93 ± 10.22	—

Disease severity and quality of life assessment

The mean mMASI was 4.25 ± 1.99 (median = 3.70; range = 0.9-9.9), reflecting predominantly mild-to-moderate disease severity. The mean Hi-MelasQoL score was 29.93 ± 10.22 (median = 30.00; range = 10-52), indicating a considerable psychosocial burden despite relatively low objective severity. Spearman’s rank correlation revealed a strong positive association between mMASI and Hi-MelasQoL (rho = 0.74; 95% CI = 0.63-0.81; p < 0.001). On univariable linear regression, each unit increase in mMASI was associated with an increase of 3.73 units in Hi-MelasQoL score (β = 3.732, SE = 0.327, 95% CI = 3.084-4.380, p < 0.001; R² = 0.527; model F(1,117) = 130.105, p < 0.001).

Dermoscopic features

Dermoscopic evaluation was performed for all 119 enrolled patients. The frequency of each dermoscopic feature is presented in Table 2. Globules were the most frequently noted feature seen in 116 (97.5%) patients, followed by pseudoreticular enhancement noted in 114 (95.8%) patients (Figure 1). Perifollicular sparing (Figure 2) was observed in 66 (55.5%) patients, telangiectasias in 61 (51.3%) patients (Figure 3), an arcuate pattern in 50 (42.0%) patients, and dots or granular pigmentation in 27 (22.7%) patients (Figure 4). 

**Table 2:** Dermoscopic features of melasma (n = 119). CI = confidence interval

Dermoscopic feature	Present, n (%)	Absent, n (%)	95% CI
Pseudoreticular enhancement	114 (95.8%)	5 (4.2%)	90.0–98.5
Globules	116 (97.5%)	3 (2.5%)	92.7–99.4
Perifollicular sparing	66 (55.5%)	53 (44.5%)	46.1–64.6
Telangiectasias	61 (51.3%)	58 (48.7%)	41.9–60.6
Arcuate pattern	50 (42.0%)	69 (58.0%)	32.9–51.6
Dots/Granules	27 (22.7%)	92 (77.3%)	15.7–31.5

**Figure 1:**
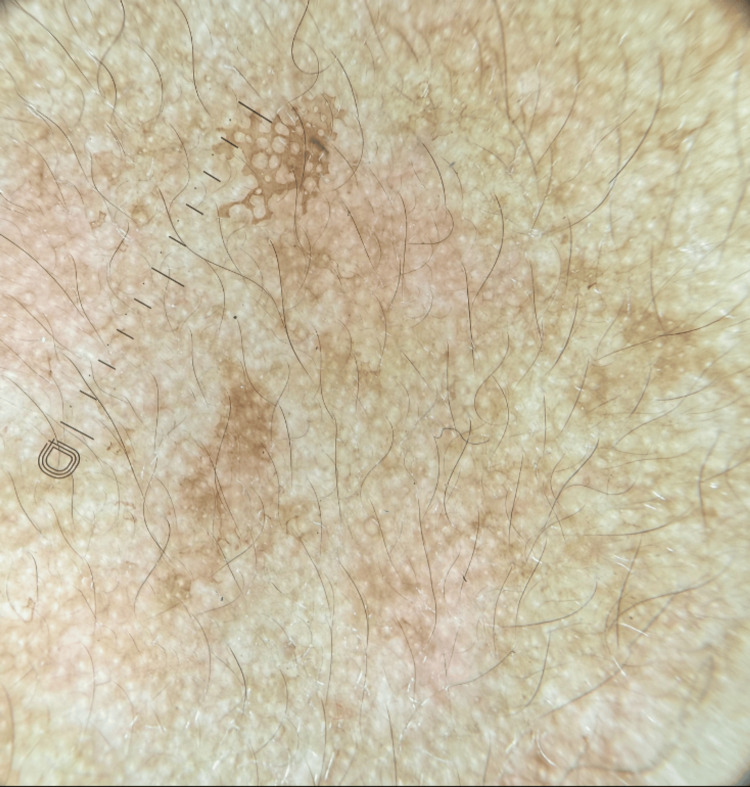
Dermoscopy of a melasma lesion revealing the reticuloglobular pattern.

**Figure 2:**
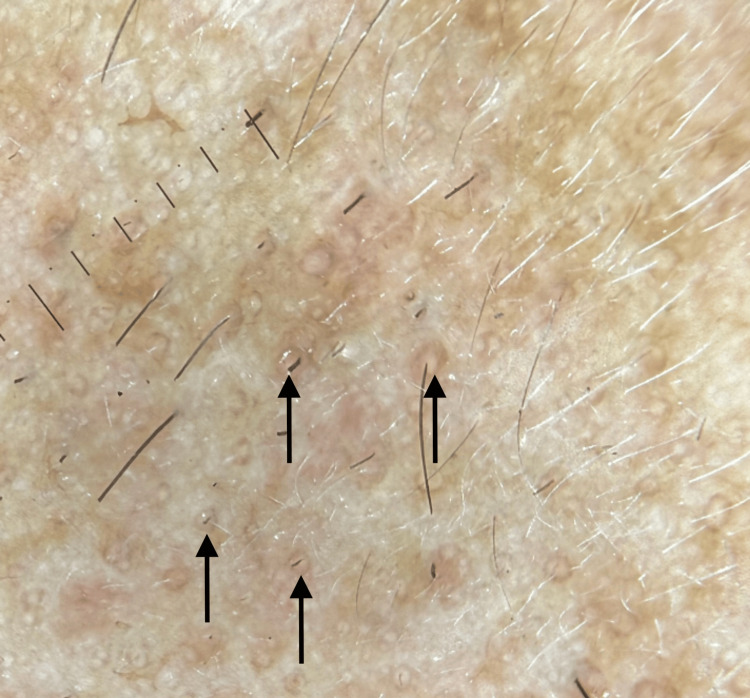
Dermoscopy of a melasma lesion revealing perifollicular sparing. Black arrows showing perifollicular sparing.

**Figure 3:**
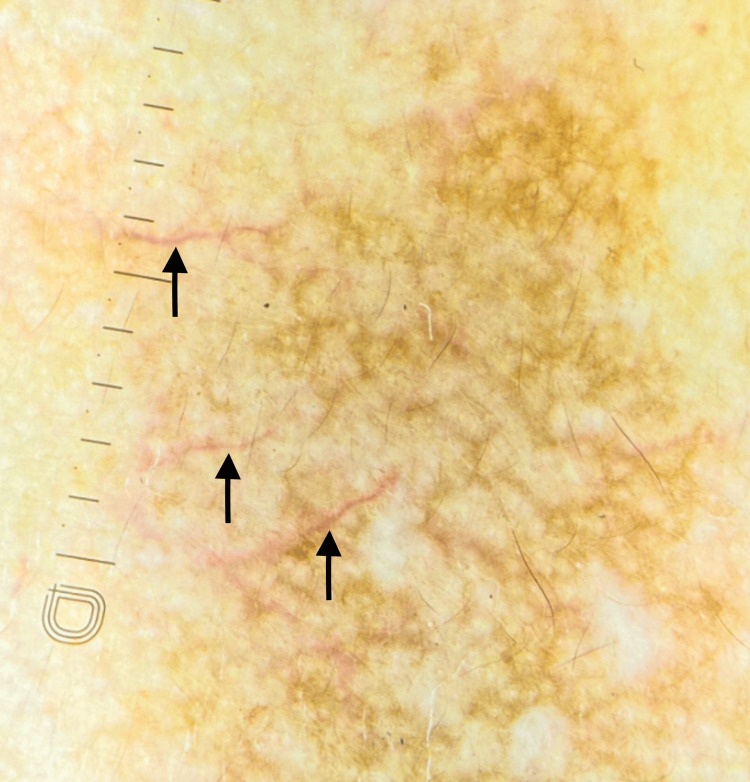
Dermoscopy of a melasma lesion revealing telangiectasias. Black arrows showing telangiectasias.

**Figure 4:**
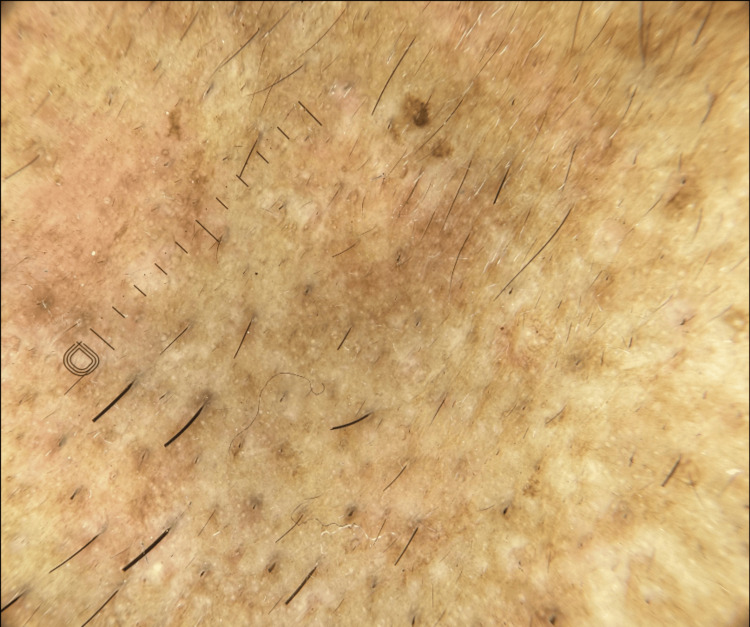
Dermoscopy of a melasma lesion revealing the dots/granular pattern.

The mean (SD) of mMASI in the group where dots/granular pattern was noted was 4.92 (1.83). The mean (SD) of mMASI in the group where dots/granular pattern was not noted was 4.05 (2.00). The median (IQR) of mMASI imMASI in the group where dots/granular pattern was noted was 5.3 (3.65-5.7). The median (IQR) of mMASI in the group where dots/granular pattern was not noted was 3.6 (2.4-5.4). The mMASI in the group where dots/granular patterns were noted ranged from 1.8 to 8.7. The mMASI in the group where dots/granular patterns were not noted ranged from 0.9 to 9.9. There was a significant difference between the two groups in terms of mMASI (W = 1590.000, p = 0.027), with the median mMASI being higher in the group where dots/granular pattern was noted.

## Discussion

The present cross-sectional study provides a comprehensive dermoscopic characterization of melasma in a North Indian cohort, with findings that align closely with, and in several respects extend, those reported in prior Indian and global studies.

Globules seen in 116 (97.5%) patients and pseudoreticular enhancement in 114 (95.8%) patients emerged as the near-universal dermoscopic hallmarks of melasma in our cohort, making them the most diagnostically consistent features. These findings are in concordance with those of Chatterjee and Neema [7], who reported a reticuloglobular pattern in 83% of Indian patients with melasma and identified it as the most characteristic dermoscopic presentation. Similarly, Kaur et al. [9] documented an accentuated pseudoreticular network in 88% and a reticuloglobular pattern in 81% of cases in another Indian study. The reticuloglobular pattern is believed to reflect the anatomical distribution of melanin within the basal layer and along the dermoepidermal junction; the globular component corresponds to focal deposits of melanin-laden keratinocytes or melanophages, while the pseudoreticular network reflects uniform basal layer hypermelanosis interrupted at follicular ostia [7,9].

Similar findings were reported by Bostan and Cakir [12], who observed a brown reticular pseudonetwork in 73.3% of lesions (173/236), and by Rattananukrom et al. [13], who noted an irregular pigment network in 92% of melasma patients (46/50).

Likewise, Solanki et al. [14] reported accentuated pseudopigment network as the most common dermoscopic finding in melasma, and Sonthalia et al. [15] described a diffuse light-to-dark brown pseudoreticular network as the fundamental dermoscopic architecture of melasma. Navya and Pai [16] described a brown reticuloglobular network predominantly in epidermal melasma and a patchy brown-grey reticuloglobular network in mixed melasma.

Perifollicular sparing, observed in 55.5% of our patients, represents a characteristic dermoscopic finding in melasma attributed to the relative paucity of melanocytes at follicular infundibula. This feature also serves as a useful marker to differentiate melasma from exogenous ochronosis, in which the follicular openings are obliterated by amorphous pigmented material [17]. The prevalence of perifollicular sparing in our study is broadly consistent with published literature, though frequencies vary depending on the proportion of epidermal versus mixed disease in individual cohorts.

Telangiectasias were identified in 51.3% of patients in the present study, a finding that closely parallels the 52% reported by Kaur et al. and somewhat exceeds the 33% reported by Chatterjee and Neema [7,9]. A similar observation was documented by Bostan and Cakir [12], who reported increased vascularity and telangiectasia in 51.7% of lesions (122/236). Amatya also found telangiectasia to be significantly associated with melasma [18].

The high prevalence of vascular structures in melasma may support the emerging paradigm of a significant vascular component in its pathogenesis, with evidence of increased dermal vascularity and elevated vascular endothelial growth factor (VEGF) expression in lesional skin reported in a few other studies [19]. Importantly, the detection of telangiectasias on dermoscopy may carry direct therapeutic relevance: the presence of prominent vascular patterns may serve as a caution against prolonged use of potent topical corticosteroids, which may exacerbate dermal atrophy and vasodilation, and may inform the selection of agents with anti-vascular properties [7,9]. Vascular-targeted therapies such as the pulsed-dye laser and tranexamic acid, the latter of which suppresses arachidonic acid-mediated melanocyte stimulation and reduces VEGF expression, may be particularly suitable in patients with prominent telangiectatic patterns. In this study, however, we did not analyze the treatment responses. Further studies are needed to confirm the same.

An arcuate pattern, representing curved or comma-shaped pigmented structures thought to correspond to the acrosyringia or eccrine duct openings and photoaged dermal elastic fibers, was detected in 42% of our patients. This frequency is somewhat higher than the 17% reported by Kaur et al., though Chatterjee and Neema described arcuate and honeycomb-like patterns as part of the broader morphological spectrum of melasma in their cohort [7,9]. The higher proportion in the present series may reflect the larger fraction of patients with mixed-type melasma (43.7%), in whom more extensive photodamage and deeper pigment distribution are anticipated.

Dots and granular pigmentation were present in 22.7% of cases, comparable to the 28% reported by Chatterjee and Neema and 19% by Kaur et al. [7,9]. These punctate pigmented structures are thought to represent dermal melanophages and are considered markers of dermal or mixed melasma. Their presence is associated with a more chronic and treatment-refractory disease course, as dermal pigment cannot be effectively targeted by conventional epidermal depigmenting agents.

Limitations

This study was a single-center observational study without blinding. The absence of a control group also limits the ability to differentiate the dermoscopic features from other facial hypermelanoses.

## Conclusions

Dermoscopy is a simple, non-invasive tool that adds useful information to the clinical evaluation of melasma. In this study, globules and pseudoreticular enhancement were the most consistently observed findings, occurring in almost all patients, and may therefore be considered key dermoscopic markers of melasma in the Indian population. Other findings, such as perifollicular sparing, arcuate structures, dots, and variation in background pigmentation, further contributed to the typical dermoscopic appearance. The frequent detection of telangiectasias suggests that vascular changes may play an important role in the development and persistence of melasma, rather than being purely a disorder of pigmentation. Dermoscopy may assist in establishing the diagnosis, estimating the depth of pigmentation, detecting vascular involvement, and distinguishing melasma from other facial pigmentary disorders. Identification of predominant brown, grey, or vascular patterns can help in selecting appropriate treatment and counselling patients regarding expected response. Overall, dermoscopy is a practical bedside modality that can improve the evaluation and management of patients with melasma.
